# Nodule carbohydrate catabolism is enhanced in the *Medicago truncatula* A17-*Sinorhizobium medicae* WSM419 symbiosis

**DOI:** 10.3389/fmicb.2014.00447

**Published:** 2014-08-27

**Authors:** Estíbaliz Larrainzar, Erena Gil-Quintana, Amaia Seminario, Cesar Arrese-Igor, Esther M. González

**Affiliations:** Departamento de Ciencias del Medio Natural/Environmental Sciences, Universidad Pública de NavarraPamplona, Spain

**Keywords:** *Medicago truncatula*, *Sinorhizobium medicae*, *Sinorhizobium meliloti*, symbiosis, efficiency, nitrogen fixation, carbon metabolism

## Abstract

The symbiotic association between *Medicago truncatula* and *Sinorhizobium meliloti* is a well-established model system in the legume–*Rhizobium* community. Despite its wide use, the symbiotic efficiency of this model has been recently questioned and an alternative microsymbiont, *S. medicae*, has been proposed. However, little is known about the physiological mechanisms behind the higher symbiotic efficiency of *S. medicae* WSM419. In the present study, we inoculated *M. truncatula* Jemalong A17 with either *S. medicae* WSM419 or *S. meliloti* 2011 and compared plant growth, photosynthesis, N_2_-fixation rates, and plant nodule carbon and nitrogen metabolic activities in the two systems.* M. truncatula* plants in symbiosis with *S. medicae* showed increased biomass and photosynthesis rates per plant. Plants grown in symbiosis with *S. medicae* WSM419 also showed higher N_2_-fixation rates, which were correlated with a larger nodule biomass, while nodule number was similar in both systems. In terms of plant nodule metabolism, *M. truncatula*–*S. medicae* WSM419 nodules showed increased sucrose-catabolic activity, mostly associated with sucrose synthase, accompanied by a reduced starch content, whereas nitrogen-assimilation activities were comparable to those measured in nodules infected with *S. meliloti* 2011. Taken together, these results suggest that *S. medicae* WSM419 is able to enhance plant carbon catabolism in *M. truncatula* nodules, which allows for the maintaining of high symbiotic N_2_-fixation rates, better growth and improved general plant performance.

## INTRODUCTION

One of the most studied plant–microbe symbiosis is the one established between members of the *Leguminosae* family and soil bacteria from diverse genera collectively termed rhizobia. When compatible symbiotic partners interact, the microsymbiont is able to invade the host root hair cells, typically (but not exclusively) through infection threads, reaching the root cortex, where they are released and differentiate into nitrogen-fixing forms; the bacteroids. In such differentiated forms, bacteria express an enzyme complex, the nitrogenase, which catalyzes the reduction of atmospheric dinitrogen (N_2_) to ammonium during the highly energy-demanding process known as symbiotic N_2_-fixation. During this complex symbiotic interaction the plant provides a carbon source, mainly in the form of malate (Udvardi et al., 1988), to be used as a respiratory substrate to fuel the N_2_-fixation process (Lodwig and Poole, 2003). Symbiotic N_2_-fixation is estimated to contribute to nearly half of the global biological N_2_-fixation reactions worldwide, representing a key process for sustainable natural and agricultural systems (Gruber and Galloway, 2008).

In recent years *Medicago truncatula* (barrel medic) has been one of the model legume species most widely studied by the symbiotic community (Barker et al., 1990; Cook, 1999). The development of mutant collections (Tadege et al., 2008; Calderini et al., 2011), optimization of transformation techniques (Boisson-Dernier et al., 2001) and availability of its genome sequence (Young et al., 2011) have greatly contributed to progress in the field.

So far at least two *Sinorhizobium* [renamed *Ensifer* (Young, 2003)] species have been described to nodulate *Medicago* spp: *Sinorhizobium meliloti* and* S. medicae* (Rome et al., 1996a). Although *M. truncatula* is able to establish N_2_-fixing symbiosis with both symbionts, most plant molecular biology studies have been carried out using the sequenced *S. meliloti* 1021 strain (Galibert et al., 2001). In recent years, however, the suitability of the *M. truncatula*–*S. meliloti* model has been questioned based on evidences that suggest that N_2_-fixation in this model is only partially effective (Moreau et al., 2008; Terpolilli et al., 2008). Instead, *S. medicae* WSM419, for which genomic sequence is also available (Reeve et al., 2010), has been suggested as a more efficient symbiont for *M. truncatula* (Terpolilli et al., 2008).

Phylogenetic analysis has shown that *S. meliloti* and *S. medicae* form a tight cluster within the *Sinorhizobium* group (Gaunt et al., 2001). Furthermore, application of several molecular markers to genetically analyze this relationship suggests that *S. medicae* was originated from an ancestral* S. meliloti* population (Biondi et al., 2003). Nowadays, these rhizobial species can be differentiated both at the phenotypic and genotypic level: *S. meliloti* is more specific for the tetraploid *M. sativa* and is preferentially found in alkaline or neutral soils, while *S. medicae* prefers diploid *Medicago* species such as *M. truncatula* and is predominantly found in moderately acid environments (Biondi et al., 2003; Garau et al., 2005). These host and environment preferences may have been a consequence of the various interspecific horizontal gene transfers that occurred during species diversification (Bailly et al., 2007; Epstein et al., 2014).

Nevertheless, to date, the physiological mechanisms underlying the higher symbiotic efficiency in the *M. truncatula* A17*–S. medicae* WSM419 association remain largely unknown. Comparative genomic studies of multiple *S. meliloti* and *S. medicae* strains have shed some light, suggesting that differences in gene content between the two species, particularly in genes involved in sulfur assimilation, conjugation and secretion, can be related to the differential symbiotic interaction and N_2_-fixation efficiency (Sugawara et al., 2013). Understanding which are the factors that underpin N_2_-fixation efficiency in legumes has potentially profound implications for sustainable agricultural systems and the environment.

In the current work, we analyzed the differences at the physiological and metabolic levels between the currently established model *M. truncatula–S. meliloti* and the more efficient *M. truncatula–S. medicae* symbiosis. We hypothesized that plant nodule metabolism may be enhanced in the *S. medicae* symbiosis compared to less efficient strains. To test this hypothesis, two sets of *M. truncatula* Jemalong A17 plants were grown under symbiotic conditions either with *S. meliloti* 2011 or *S. medicae* WSM419. Plant growth parameters, photosynthesis, N_2_-fixation, and plant nodule carbon and nitrogen metabolic activities were determined. Results presented here show that *S. medicae* WSM419-derived nodules generate a stronger sink in the plant, through the activation of sucrose-hydrolyzing enzymes. This allows the maintenance of high N_2_-fixation rates, increased nodule growth, and, therefore, a generally improved plant performance.

## MATERIALS AND METHODS

### GROWTH CONDITIONS

*Medicago truncatula* Gaertn cv. Jemalong A17 plants were grown in 1-L pots with a mixture of perlite:vermiculite (2:5, v/v) as substrate under controlled environmental conditions (14 h day/10 h night; 450 μmol m^-2^ s^-1^ light intensity; 22°C/16°C day/night temperature; 60–70% relative humidity). After germination, plantlets were separated into two sets: one was inoculated with *S. meliloti* strain 2011 (Meade and Signer, 1977) and the other was inoculated with *S. medicae* strain WSM419 (Rome et al., 1996b). Bacterial cultures were grown on a rotary shaker (175 rpm) at 28°C for 48 h in yeast extract mannitol broth containing (*g* L^-1^) K_2_HPO_4_ (0.5), 0.2 MgSO_4_⋅7H_2_O, NaCl (0.1), mannitol (10), and yeast extract (0.4), pH adjusted to 6.8, to an OD_600_ of 0.7–0.8, which corresponds to ∼3 × 10^8^ cells (Vincent, 1970). 1 ml of the cultures was inoculated onto each seedling at sowing.

Plants were watered with a nutrient solution containing (values in mg L^-1^): MgSO_4_⋅7H_2_O (493), K_2_SO_4_ (279), K_2_HPO_4_ (145), CaCl_2_ (56), KH_2_PO_4_ (23), EDTA-Fe (17), H_3_BO_3_ (1.43), CaSO_4_⋅2H_2_O (1.03), MnSO_4_⋅7H_2_O (0.77), ZnSO_4_⋅7H_2_O (0.22), CoCl_2_⋅6H_2_O (0.12), CuSO_4_⋅5H_2_O (0.08), NaMoO_4_⋅2H_2_O (0.05). For the first 3 weeks, 0.25 mM ammonium nitrate was added to the nutrient solution. Eight weeks after planting, symbiotic N_2_-fixation was measured, nodules collected, divided into aliquots, frozen in liquid N_2_ and stored at -80°C for analytical determinations. Two nodule aliquots per plant were used for nodule number estimation based on total nodule weight. Shoots and roots were weighed for fresh weight (FW) determinations and, subsequently, oven-dried at 80°C for 48 h before dry weight (DW) was measured.

### NITROGEN FIXATION AND CHLOROPHYLL CONTENT DETERMINATIONS

Symbiotic N_2_-fixation was measured in intact plants as apparent nitrogenase activity (ANA). H_2_ evolution from sealed roots systems was measured in an open flow-through system under N_2_:O_2_ (79%:21%, v/v) according to Witty and Minchin (1998) using an electrochemical H_2_-sensor (Qubit System, Canada).

Photosynthesis was determined in the apical leaves with an open system mode (model LC pro+; ADC BioScientific Ltd., Great Amwell, UK) using an ADC PLC-7504 leaf chamber. To estimate leaf chlorophyll content a Minolta SPAD-502 system was employed (Konica Minolta Sensing Europe BV, UK).

### NODULE PROTEIN EXTRACTION AND ENZYMES ASSAY

Nodules (100 mg FW) were homogenized in a mortar and pestle with 500–600 μL of extraction buffer (50 mM 3-(*N*-morpholino)propanesulfonic acid (MOPS), 5 mM MgCl_2_, 20 mM KCl, 1 mM EDTA, 20% polyvinylpolypyrrolidone, pH 7) where 1.5 mg mL^-1^ of DTT, 0.7 μL mL^-1^ of β-mercaptoethanol and 20 μL mL^-1^ plant protease inhibitor cocktail (Sigma-Aldrich) were freshly added. Homogenates were centrifuged at 12,000 *g* and 4°C for 15 min and supernatants were collected as nodule plant fractions. The nodule plant fraction was desalted using Bio Gel P6DG columns (Bio-Rad) equilibrated with 250 mM MOPS (pH 7), 100 mM KCl and 25 mM MgCl_2_. The desalted extract was used to measure the following enzyme activities according to Gonzalez et al. (1998): sucrose synthase (EC 2.4.1.13), alkaline invertase (EC 3.2.1.26), NADH-dependent glutamate synthase (GOGAT; EC 1.4.1.14), and aspartate aminotransferase (AAT; EC 2.6.1.1). The protein content in crude and desalted extracts was quantified using a Bradford-based dye-binding assay (Bio-Rad) employing bovine serum albumin as standard.

### CARBOHYDRATE AND STARCH DETERMINATION

100 mg-FW nodule aliquots were extracted in 80% (v/v) ethanol and ultrasonicated in a water bath system. After sonication, samples were centrifuged at 7,500 *g* and 4°C for 5 min and supernatants were collected. These steps were repeated three times. Afterward the supernatants were dried in a Turbovap LV evaporator (Zymark Corp, Hopkinton, MA, USA) and soluble compounds were redissolved in 1 mL distilled water, homogenized and stored at -20°C. The ethanol-insoluble residue was extracted for starch determination as in Macrae (1971). Carbohydrates were analyzed by high-performance capillary electrophoresis (Warren and Adams, 2000) using 10 mM benzoate (pH 12) containing 0.5 mM myristyltrimethylammonium bromide as a buffer under the following conditions: -15 kV potential, 50 μm-internal diameter and 30/40.2 cm-long capillary tube, indirect UV detection at 225 nm.

### STATISTICAL ANALYSIS

All data are reported as mean ± standard deviation of *n* = 5 independent measurements. Statistical analysis was conducted using Student’s *t*-test and *p*≤ 0.05 was considered as statistically significant. The homogeneity of variances was tested using Levene’s test.

## RESULTS

**FIGURE 1:**
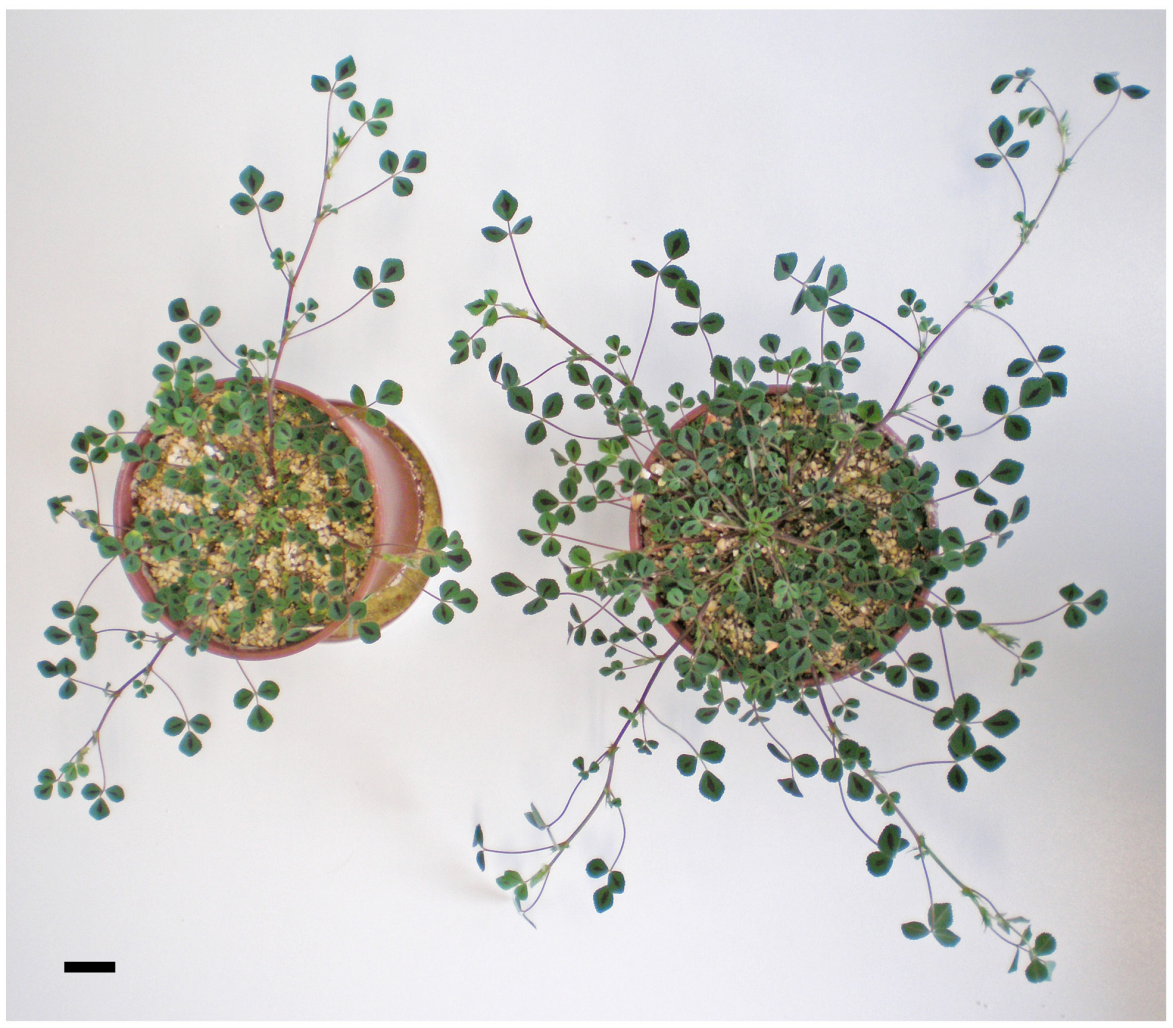
***Medicago truncatula* cv. Jemalong A17 plants 8 weeks after inoculation with either *Sinorhizobium meliloti* 2011 (left) or *Sinorhizobium medicae* WSM419 (right).** Scale bar = 2 cm.

In general terms, *M. truncatula* plants inoculated with *S. medicae* WSM419 outperformed those inoculated with *S. meliloti* 2011. Total plant biomass in the *M. truncatula–S. medicae* system was more than two-fold higher than when using the *S. meliloti* strain and the difference was most notable for shoots (**Figure 1**; **Table 1**). Plants inoculated with the *S. medicae* strain maintained a 1:1 shoot-to-root ratio, while this declined to ∼3:4 in plants inoculated with *S. meliloti* 2011 (**Table 1**).

**Table 1:** Plant biomass.

Biomass (*g* FW)	*M. truncatula–S. meliloti* 2011	*M. truncatula–S. medicae* WSM419
Shoot	9.10 ± 1.81	26.48 ± 1.00*
Root	12.85 ± 2.55	25.70 ± 2.73*
Total plant	22.40 ± 4.43	52.95 ± 2.66*
Shoot:root ratio	0.71 ± 0.02	1.08 ± 0.13*

M. truncatula plant biomass values when grown in symbiosis with *S. meliloti* 2011 or *S. medicae* WSM419. Values are mean ± standard deviation of five biological replicates. An asterisk (*) denotes significant differences (Student’s *t*-test at *p* ≤ 0.05). FW = fresh weight.

**FIGURE 2:**
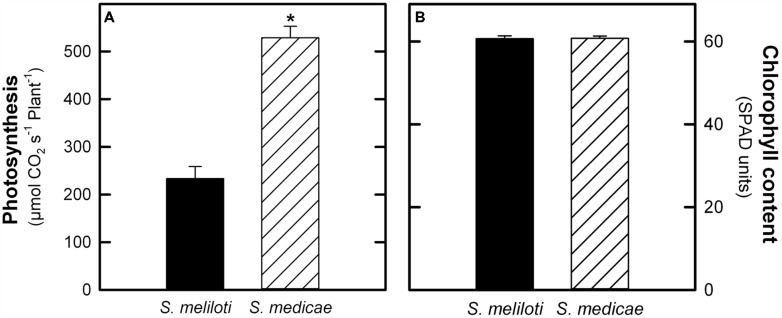
**Photosynthesis rates **(A)** and chlorophyll content **(B)** in *M. truncatula* inoculated with either *S. meliloti* 2011 or *S. medicae* WSM419.** Values represent mean ± standard deviation (*n* = 5). An asterisk (*) denotes significant differences (Student’s *t*-test at *p* ≤ 0.05).

Regarding photosynthetic CO_2_ assimilation, *M. truncatula*–*S. medicae* plants showed a 55.8% increase in photosynthesis when expressed on a plant basis (**Figure 2A**). However, when expressed on a leaf area basis, *M. truncatula*–*S. meliloti* showed higher photosynthetic rates (86.37 ± 2.09 μmol CO_2_ s^-1^ cm^-2^) compared to *S. medicae*-inoculated plants (67.19 ± 2.43 μmol CO_2_ s^-1^ cm^-2^). These higher photosynthetic rates were, however, not correlated with increased leaf chlorophyll content values, with both plant systems presenting similar values (**Figure 2B**).

**FIGURE 3:**
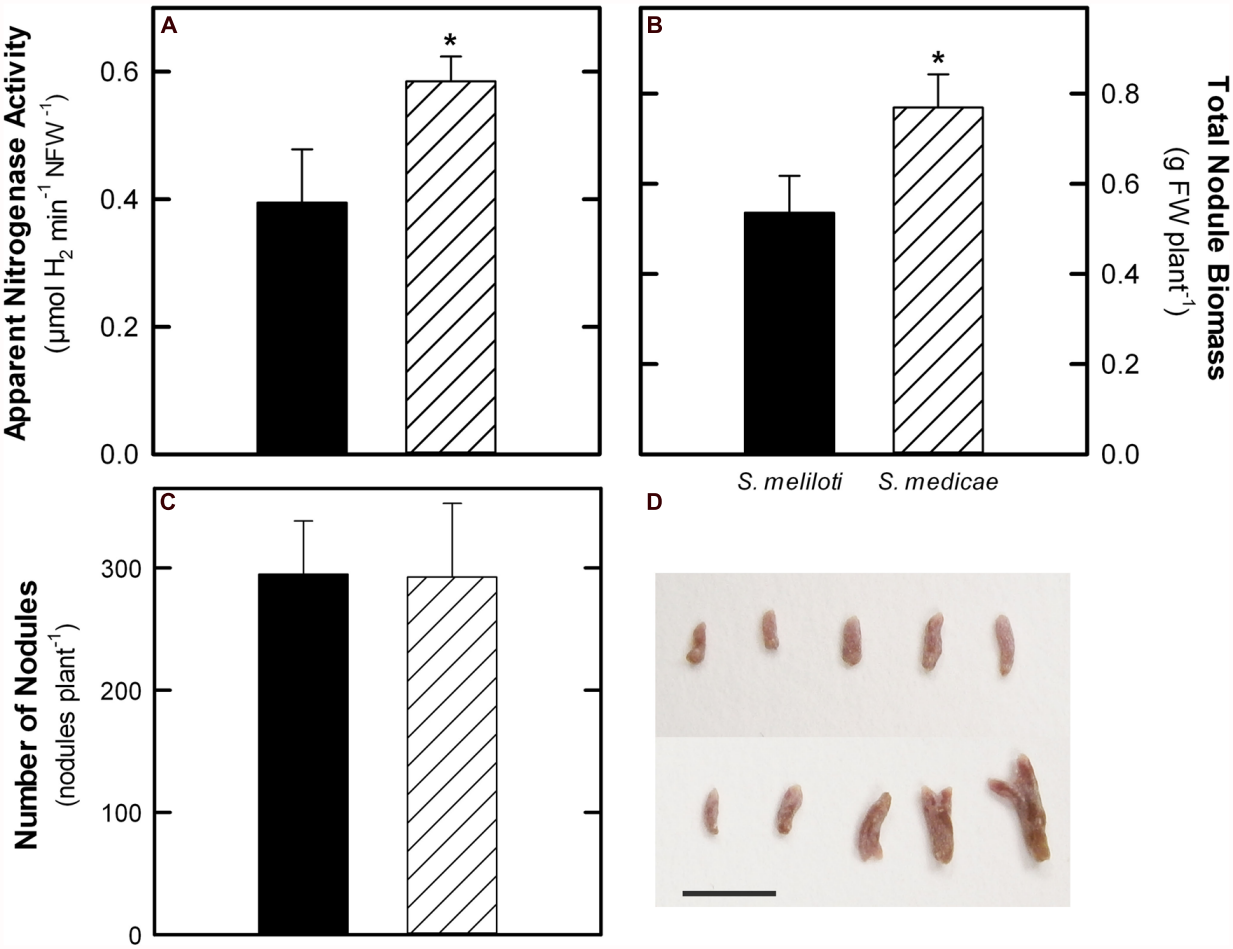
**N_**2**_-fixation rates measured as apparent nitrogenase activity (ANA, **A**), total nodule biomass **(B)**, nodule number **(C)** in *M. truncatula* plants inoculated with either *S. meliloti* 2011 or *S. medicae* WSM419. D**, representative image of nodules sampled from plants inoculated with *S. meliloti* 2011 (top) or *S. medicae* WSM419 (bottom). Scale bar = 500 μm. Values represent mean ± standard deviation (*n* = 5). An asterisk (*) denotes significant differences (*p* ≤ 0.05).

To accurately estimate the rates of N_2_-fixation, ANA was measured as H_2_ evolution in intact plants (Witty and Minchin, 1998). The *M. truncatula*–*S. medicae* symbiosis showed increased N_2_-fixation values both when expressed on a plant (+57%) and nodule FW basis (**Figure 3A**). Plants inoculated with *S. medicae* showed higher nodule biomass (**Figure 3B**), although the number of root nodules was similar in both cases (**Figure 3C**). The increase in nodule biomass was, therefore, correlated with higher biomass per nodule. Plants inoculated with the *S. medicae* strain presented larger and more frequently bifurcated nodules compared to plants inoculated with the *S. meliloti* strain (**Figure 3D**). Furthermore, the plant fraction of *M. truncatula–S. medicae* nodules showed a significantly higher protein content than that of nodules infected with *S. meliloti* (25.18 ± 3.32 vs. 20.53 ± 3.58 mg protein g FW^-1^, mean ± standard deviation, respectively).

**FIGURE 4:**
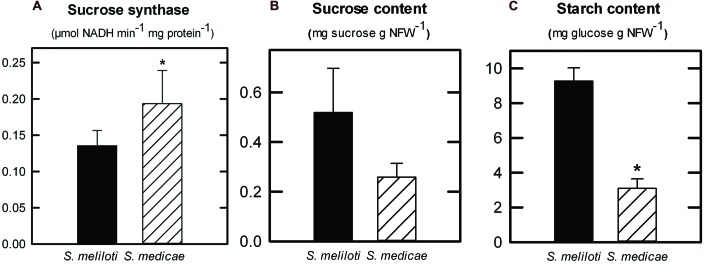
**Sucrose synthase **(A)** enzymatic activity in *M. truncatula* nodules inoculated with either *S. meliloti* 2011 or *S. medicae* WSM419.** Values are given in μmol NADH min^-1^ mg plant protein^-1^ and represent mean ± standard deviation (*n* = 5). Sucrose **(B)** and starch content **(C)** in *M. truncatula* nodules inoculated with either *S. meliloti* 2011 or *S. medicae* WSM419. Values (in mg g NFW^-1^) represent mean ± standard deviation of five biological replicates. An asterisk (*) denotes significant differences (*p* ≤ 0.05).

To better understand the metabolic differences in nodules following inoculation with the two microsymbionts, we measured the activity of the two main sucrose-degrading enzymes in nodules, sucrose synthase and alkaline invertase, as well as the activity of two key enzymes involved in ammonium assimilation, GOGAT and AAT. In both systems the specific activity of sucrose synthase was on average more than 25-fold higher than that of alkaline invertase (data not shown). Comparing the activity levels across systems, only sucrose synthase showed a significant increase in *S. medicae*-infected nodules (**Figure 4A**). In terms of nodule nitrogen metabolism, neither GOGAT nor AAT activities showed significantly different rates when comparing the two inoculants (data not shown).

Given that nodule sucrose catabolism was found to be more active in the *M. truncatula–S. medicae* symbiosis, the main carbon metabolites in nodules were quantified; sucrose and starch (**Figures 4B,C**). As a general trend, *S. medicae*-infected nodules presented lower levels of carbohydrates compared to those infected by the *S. meliloti* strain, with significant differences found in terms of starch content (**Figure 4C**).

## DISCUSSION

The efficiency of a legume–*Rhizobium* symbiosis is usually evaluated by comparing plant growth parameters (e.g., biomass, N content) of inoculated versus N-fed plants. These types of study, mostly analyzed from the bacterial perspective, have demonstrated that symbiotic efficiency varies depending upon the specific bacterial strain used (Miller and Sirois, 1982; Mhadhbi et al., 2005; Parra-Colmenares and Kahn, 2005; Heath and Tiffin, 2007; Rangin et al., 2008; Terpolilli et al., 2008; Oono and Denison, 2010). However, the plant contribution to these variable efficiencies has received much less attention.

In this work, we analyzed the effectiveness of the symbiosis of *M. truncatula* A17 with two *Sinorhizobium* strains, *S. meliloti* 2011 and *S. medicae* WSM419, with special emphasis on understanding the main differences at the nodule metabolic level. Under our experimental growth conditions, *M. truncatula* plants grown almost exclusively on fixed N upon inoculation with *S. meliloti* 2011 did not show symptoms of N deficiency, presenting leaf chlorophyll contents comparable to those of plants inoculated with the *S. medicae* strain (**Figure 2B**). We did, however, observe a general outperformance of plants inoculated with the *S. medicae* strain in terms of plant biomass (**Figure 1**; **Table 1**), photosynthesis per plant (**Figure 2A**) and N_2_-fixation rates (**Figure 3A**). Interestingly, this improved fixation performance was correlated with a larger biomass per nodule, leading to a higher total nodule biomass per plant, but not to increased nodule number (**Figure 3**).

Nodules are strong sink tissues due to the high-energy demand that symbiotic N_2_-fixation represents for the plant (Silsbury, 1977; Schuize et al., 1999). These high-energy requirements are met by allocating photoassimilates from the aerial part to nodules, mostly in the form of sucrose, where they are hydrolyzed by either sucrose synthase or alkaline invertase (Morell and Copeland, 1984; Flemetakis et al., 2006). Sucrose synthase is considered to be primarily responsible for sucrose metabolism in mature nodules and its role has been shown to be essential for symbiotic N_2_-fixation in legumes (Gordon et al., 1999; Baier et al., 2007; Horst et al., 2007), while alkaline invertase appears to have a secondary role (Welham et al., 2009). In this study, the predominant role of sucrose synthase as the main sucrose-degrading enzyme in nodules was corroborated, showing a significantly higher specific activity than that of alkaline invertase in both symbiotic systems (>20-fold higher in average). Nodules from plants inoculated with the more efficient *S. medicae* strain showed higher sucrose synthase activity compared to *S. meliloti* 2011 nodules (**Figure 4A**). Furthermore, *S. medicae* WSM419-inoculated plants maintained nodule starch at significantly lower levels compared to those inoculated with the *S. meliloti* strain (**Figure 4C**), despite the higher photosynthetic rates of the former (**Figure 2A**). This inverse correlation between symbiotic efficiency and starch accumulation has been similarly observed in alfalfa plants when inoculated with a fix - strain (Aleman et al., 2010). Indeed, in non-fixing alfalfa nodules, the products from sucrose breakdown are re-directed to starch biosynthesis due to the lower energy demand. Taken together, these results suggest that *S. medicae* WSM419 activates plant carbon catabolic reactions in nodules to keep up with the high nitrogenase demand for ATP and, as a consequence, they become stronger metabolic sinks in the plant (Sung et al., 1989). This positive feedback keeps N_2_-fixation rates high, promoting plant growth and, therefore, increasing the plant photosynthetic capacity. A similar mechanism has been described when bacteroid respiration is enhanced in nodules by the overexpression of a cytochrome oxidase (Soberon et al., 1999; Silvente et al., 2002; Talbi et al., 2012).

Despite the differences in N_2_-fixation rates, plants inoculated with the *S. meliloti* strain did not show significant differences in terms of nodule number (**Figure 3C**). Differences were, however, found in the plant protein fraction of nodules, most likely related to the metabolic activation discussed above. It is interesting, though, that these differences are mostly observed at the level of carbon metabolism, while the specific activity of enzymes involved in N assimilation did not differ significantly when the two symbiotic systems were compared (data not shown).

In conclusion, results presented here suggest that at least one of the factors contributing to the higher effectiveness of the *M. truncatula–S. medicae* WSM419 symbiosis is the activation of plant carbon catabolism in nodules, which allows the maintenance of high N_2_-fixation rates and, ultimately, leads to an improved plant performance. In agreement with previous studies (Moreau et al., 2008; Terpolilli et al., 2008), the use of *S. medicae* WSM419 as the partner of choice for *M. truncatula* symbiotic studies is highly recommended.

## Conflict of Interest Statement

The authors declare that the research was conducted in the absence of any commercial or financial relationships that could be construed as a potential conflict of interest.
